# GLYI and D-LDH play key role in methylglyoxal detoxification and abiotic stress tolerance

**DOI:** 10.1038/s41598-018-23806-4

**Published:** 2018-04-03

**Authors:** Muskan Jain, Preeti Nagar, Ayush Sharma, Rituraj Batth, Sakshi Aggarwal, Sumita Kumari, Ananda Mustafiz

**Affiliations:** 1 Faculty of Life Sciences and Biotechnology, Laboratory of Plant Molecular Biology, South Asian University, Akbar Bhawan, Chanakyapuri, New Delhi, 110021 India; 2 School of Biotechnology, Sher-e-Kashmir University of Agricultural Sciences and Technology, Jammu, 180009 India

## Abstract

Methylglyoxal(MG) is a potent cytotoxin that is produced as a byproduct of various metabolic reactions in the cell. The major enzymes for MG detoxification are Glyoxalase I(GLYI), Glyoxalase II(GLYII) and D-lactate dehydrogenase(D-LDH). These three enzymes work together and convert MG into D-pyruvate, which directly goes to TCA cycle. Here, a comparative study of the ability of MG detoxification of these three enzymes has been done in both *E. coli* and yeast. Ectopic expression of these three genes from Arabidopsis in *E. coli* in presence of different abiotic stress revealed the contribution of each of these genes in detoxifying MG. Yeast mutants of MG detoxification enzymes were also grown in different stress conditions to record the effect of each gene. These mutants were also used for complementation assays using the respective MG detoxifying genes from Arabidopsis in presence of various stress conditions. The MG content and the corresponding growth of cells was measured in all the bacterial as well as yeast strains. This study reveals differential contribution of MG detoxification enzymes in mitigating MG levels and alleviating stress in both prokaryotes as well as eukaryotes. GLYI and D-LDH were found to be key enzymes in MG detoxification under various abiotic stresses.

## Introduction

Methylglyoxal (MG) is a three carbon metabolite which is present in all the organisms from prokaryotes to eukaryotes. It is produced as a by-product of various metabolic reactions such as glycolysis, lipid peroxidation, protein degradation and photosynthesis. MG has been found to function as a signaling molecule in bacteria^1^, yeast^2–6^, animals^7–15^ and plants^16–18^. MG also acts as a stress signal molecule in plant system and triggers a response by inducing several protein kinases and transcription factors^19^. But this signaling function is only at low concentrations. At higher concentration, MG is detrimental for the cell and the whole system as it reacts with major macromolecules including DNA, RNA^20^, proteins^21^ and also inhibits cell proliferation^22^. MG levels increase by 2–6 folds in response to abiotic stress^23^. The increase in MG level due to stress has been reported in animals, mammals, yeast and bacterial systems^24,25^ and also in plants^23^. This excessive MG accumulation in plant cells under stress can inhibit cell proliferation and cause the inactivation and degradation of proteins, inactivation of antioxidant defenses, leading to disruption of many cellular functions^26^ ultimately resulting in decreased growth and yield of the plants; more than 70% reduction in crop production has been attributed to poor environmental conditions^27^. Since, stress leads to increased level of glycolysis, hence spontaneous production of MG via glycolysis is an unavoidable consequence^28^ and therefore, the only way to combat the toxic effects is to detoxify MG.

Glyoxalase pathway is the major system for MG detoxification in all the organisms including bacteria, yeast, humans, plants and animals. It comprises of two enzymes, Glyoxalase I (GLYI) which converts MG to S-D-lactoylglutathione (SLG) and Glyoxalase II (GLYII) which converts SLG to D-lactate^29^. Another enzyme Glyoxalase III (GLYIII) has been discovered which directly converts MG to D-lactate in a single step, without using GSH or any other cofactor^30^. Recently, another enzyme, D-lactate dehydrogenase (D-LDH) has been linked with MG detoxification which catalyzes break down of end product of glyoxalase system, D-lactate, into D-pyruvate which enters into TCA cycle for energy production^31,32^. Apart from glyoxalase system, certain other enzymes have been found to metabolize MG. MG has two functional groups, which can be either oxidized or reduced, due to which it acts as a substrate for various oxidoreductase and dehydrogenase enzymes^33^. Various aldo-keto reductases and dehydrogenases have been identified in different species^34–37^. The aldo-keto reductases use NADH or NADPH to reduce MG to acetol, lactaldehyde or pyruvate^33,37–39^. Methylglyoxal reductase catalyzes the reduction of 2-oxoaldehydes to the corresponding 2-hydroxyaldehydes and then converts aldehydes to alcohol^40^. A NADH and NADPH dependent methylglyoxal reductase has been identified in *E.coli* which converts methylglyoxal directly to acetol (Misra *et al*. 1996). The overexpression of these aldo-keto reductases in plants have been found to confer tolerance to various stresses. Another enzyme, MG dehydrogenase converts MG into pyruvate.

**Figure 1:**
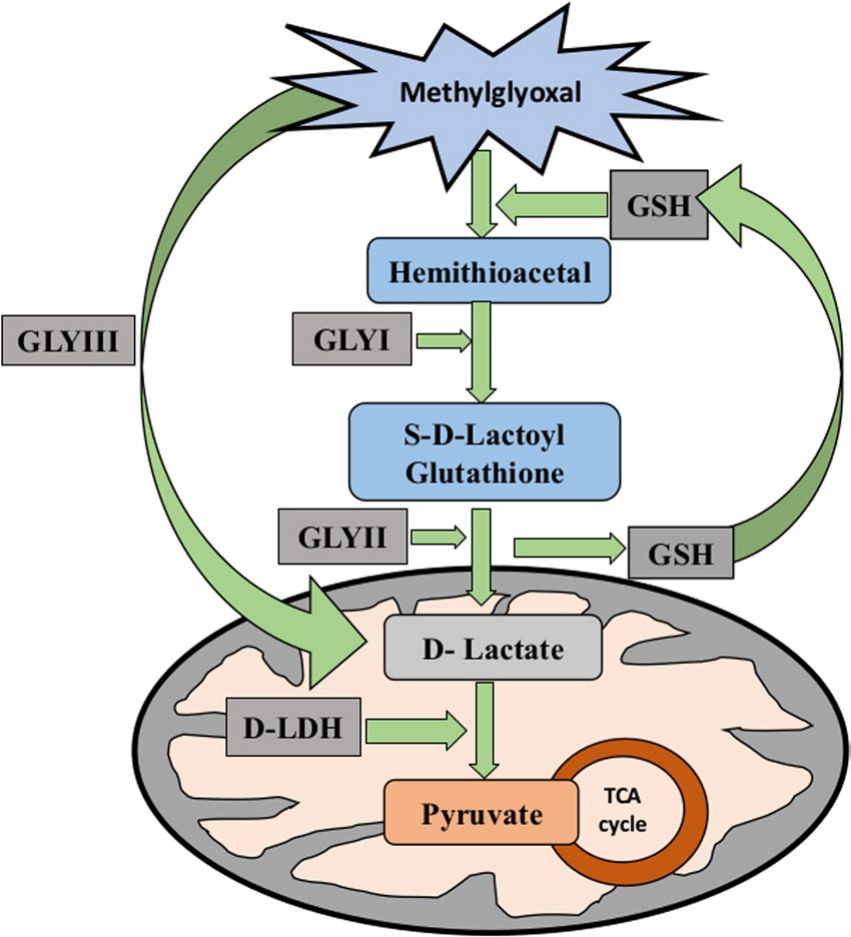
Methylglyoxal detoxification pathway: MG is a toxic metabolite produced in the cell. The detoxification of MG is carried out by various pathways; major one being glyoxalase system that consists of two enzymes, Glyoxalase I and II. Glyoxalase I enzyme converts MG into S-D-lactoyl glutathione which is converted to D-lactate by Glyoxalase II. Glyoxalase III converts MG directly into D-lactate without using any cofactor. Further another enzyme, D-LDH converts this D-lactate into D-pyruvate which goes to TCA cycle. Thus, the toxic MG is diverted to produce energy for the cell.

MG detoxification mainly involves three enzymes GLYI, GLYII and D-LDH (Fig. 1). In the present study, these three enzymes have been compared for their role in MG detoxification. All these enzymes are a part of multimember family in Arabidopsis. The most active and efficient member of each family, AtGLYI (AT1G08110), AtGLYII (AT3G10850) and AtD-LDH (AT5G06580), was selected and a comparative study was undertaken^31,41,42^. AtGLYI, AtGLYII and AtD-LDH were cloned in pET28a, bacterial expression vector and pYES2, yeast expression vector. The heterologous overexpression of these genes in bacteria and yeast provided varying degree of tolerance towards different abiotic stresses. Further, to strengthen the findings of stress tolerance assays in *E. coli* and yeast, loss of function mutants of MG detoxifying genes were analyzed for their stress mitigating ability in presence and absence of stress conditions. These mutants were also used for complementation assays using the corresponding genes w.r.t. their ability to revert the mutant phenotype. Results of stress tolerance and complementation assays were validated by measuring endogenous MG level in wild type as well as mutant cells grown in presence and absence of stress to correlate the effect of each gene in reducing MG level and congruent tolerance to abiotic stress.

## Material and Methods

### Cloning of AtGLYI, AtGLYII and AtD-LDH genes

Total RNA was isolated from fresh Arabidopsis leaf tissue using IRIS Kit (Bangalore, Genei) according to manufacturer’s instructions. The RNA was reverse transcribed using the Revert Aid H Minus first stand cDNA synthesis Kit (Fermentas Life Sciences, USA). This first strand cDNA was used to amplify AtGLYI, AtGLYII and AtD-LDH genes using Q5 DNA polymerase (NEB). The primer sequences used for cloning are listed in Supplementary Table S1. The PCR product of all three genes were cloned into blunt end pJET cloning vector (CloneJET^TM^ PCR cloning Kit, Fermentas Life Sciences, USA). Once the correct sequence of all the three genes was confirmed, they were subcloned into pET28a (Novagen), bacterial expression vector and pYES2 (Invitrogen), yeast expression vector, under the control of galactose inducible and glucose repressible GAL1 promoter.

### Abiotic stress tolerance assay in *E. coli*

The constructs pET28a-AtGLYI, pET28a-AtGLYII, pET28a-AtD-LDH and empty vector pET28a were transformed in BL21 cells. BL21 cells containing these constructs were grown at 37 °C till the culture reached its mid exponential phase, i.e. OD_600_ was between 0.4–0.6. Then, the cells were induced with 0.5 mM IPTG and grown for 12 hours in presence of different stressors i.e. 200 mM NaCl, 5 mM H_2_O_2_ and 0.5 mM exogenous MG. OD_600_ was measured at an interval of every 2 hours. After that, the cells were harvested and MG estimation was done. This was done in triplicates and the values obtained were averaged and used to plot graphs.

### Stress tolerance assay in yeast cells

Wild type cells of yeast strain BY4741 and mutants of MG detoxification enzymes i.e., GLYI mutant BY4741 cells (GLYIKO), GLYII mutant BY4741cells (GLYIIKO) and D-LDH mutant BY4741 cells (D-LDHKO) were transformed with empty vector pYES2. The mutants were complemented for the missing gene by transforming the pYES2 construct containing the corresponding gene from Arabidopsis. GLYIKO cells were transformed with pYES2-AtGLYI, GLYIIKO cells with pYES2-AtGLYII and D-LDH mutants with pYES2-AtD-LDH. The positive transformants were picked via Ura^−^ prototrophy. BY4741(pYES2), GLYIKO(pYES2), GLYIIKO(pYES2) and D-LDHKO(pYES2) cells were grown in Ura^−^ liquid media in presence of different abiotic stresses such as 1 M NaCl, 25 mM H_2_O_2_ and 2 mM MG. Their growth was monitored by taking OD_600_ every 5 hours till 30 hours. The cells were then harvested and MG content was measured. Similarly, growth pattern was recorded and MG levels were estimated for the mutant cells complemented with respective genes. All experiments were done in triplicates. The averaged values were used for plotting graphs and comparison.

The mutant as well as complemented yeast cells were streaked on solid Ura^−^ media plates containing either 20% glucose or 20% galactose with different concentrations of MG (0–5 mM). The plates were kept at 30 °C for 4–5 days. The growth pattern of both wild type and mutant yeast strains harboring knockouts of GLYI, GLYII and D-LDH genes was observed. BY4741(pYES2) was used as a reference control.

### MG estimation

The measurement of MG level was done by modifying the protocol described previously^43^. The cell lysate was prepared by resuspending the cell pellet in 250 μl of 5 M perchloric acid. It was incubated in ice for 15 min. The lysate was centrifuged at 11,000 g for 10 min. The supernatant obtained was neutralized using 1 M Na_2_HPO_4_. Again it was centrifuged and the supernatant obtained was transferred to a fresh tube. To 1 ml of this supernatant, 10 μl of 100 mM NaN_3_ was added. Reaction mixture containing 250 μl of 7.2 mM 1,2-diaminobenzene, 100 μl of 5 M perchloric acid and 650 μl of the final supernatant was prepared. Absorbance at 336 nm was measured immediately and this was considered as the blank reading. The reaction mixture was incubated in dark at room temperature for 3 hours and absorbance was measured at 336 nm. MG content was calculated using a standard curve. For standard curve, different concentrations of MG (2 μM–150 μM) were used. The values obtained were used to plot graphs.

## Results

### Heterologous expression of MG detoxification enzymes in *E. coli* confers tolerance to various abiotic stresses

**Figure 2:**
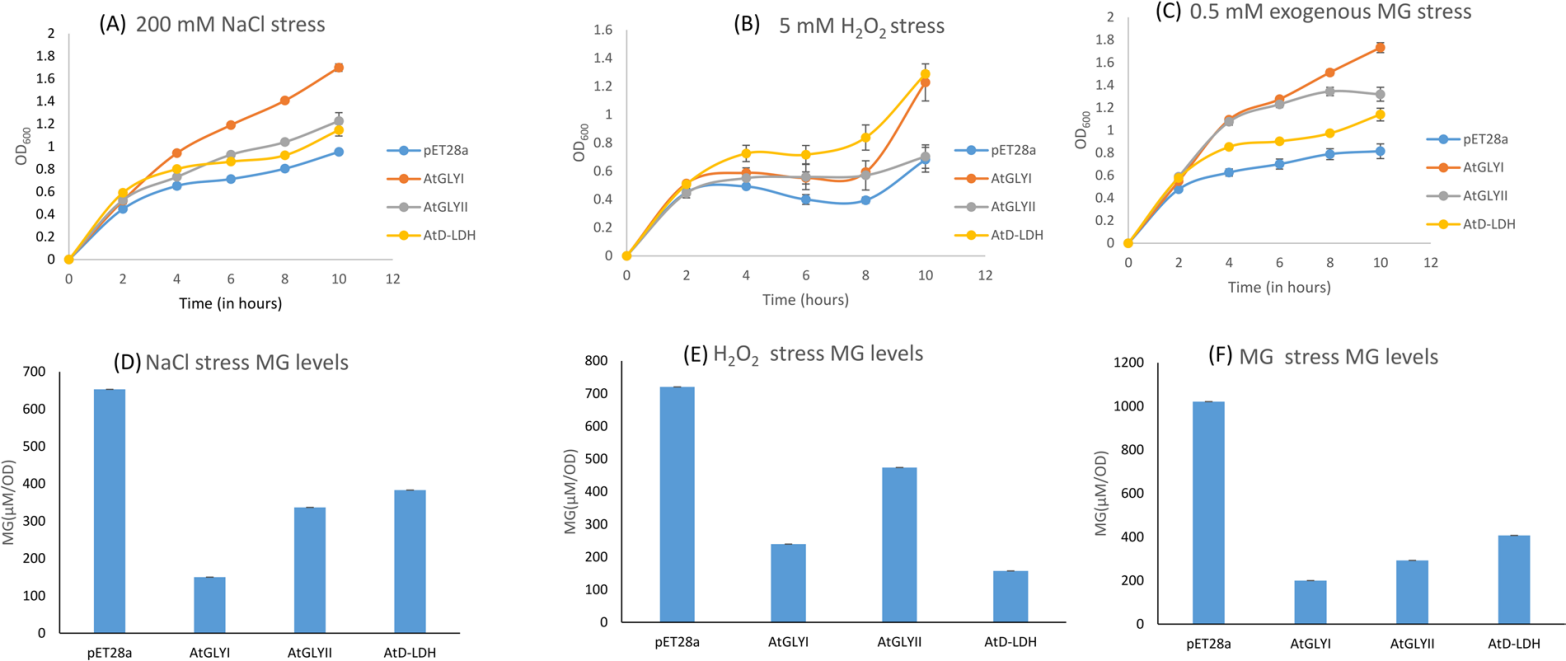
Heterologous expression of MG detoxification enzymes in *E. coli* provides varying tolerance to various abiotic stresses: The BL21 *E. coli* cells containing the constructs of MG detoxification genes (pET28a-AtGLYI, pET28a-AtGLYII and pET28a-AtD-LDH) were grown in presence of different abiotic stresses such as (**A**) salinity (200 mM NaCl), (**B**) oxidative (5 mM H_2_O_2_) and (**C**) exogenous MG (0.5 mM MG) and their growth was monitored. Cells containing empty vector were used as control.

BL21 cells transformed with pET28a-AtGLYI, pET28a-AtGLYII, pET28a-AtD-LDH and empty vector pET28a were grown in presence of different abiotic stresses and their growth was monitored spectrophotometrically. The MG level in each cell type subjected to a specific stress was estimated. In response to all the stresses under study, *E. coli* cells overexpressing Arabidopsis genes for MG detoxification enzymes grew better than the cells transformed with empty vector and also accumulated lower MG levels than the cells with empty vector. In case of salinity and exogenous MG stress, GLYI overexpressing cells grew better than GLYII overexpressing cells which in turn were better than D-LDH overexpressing cells (Fig. 2A and C). GLYI overexpressing cells had the lowest MG followed by GLYII and D-LDH overexpressing cells (Fig. 2D and F). However, in response to oxidative stress, D-LDH overexpressing cells provided more tolerance in comparison to GLYI and GLYII overexpressing cells (Fig. 2B). The pattern of MG level negatively correlated with the growth of *E. coli* cells subjected to oxidative stress. AtD-LDH expressing cells accumulated the least MG level. AtGLYI expressing cells showed more level of MG than AtD-LDH expressing cells but lesser than AtGLYII expressing cells (Fig. 2E).

### GLYI provides more tolerance in salinity stress in yeast cells

The wild type cells of yeast strain BY4741 and the yeast mutants in the same strain for GLYI, GLYII and D-LDH genes transformed with empty vector pYES2 were grown in standard media supplemented with different abiotic stresses. Thereafter the same mutants were transformed with a copy of corresponding functional MG detoxification gene from *Arabidopsis thaliana*. The ability of these genes to complement knockout of yeast MG detoxification genes was recorded by observing their growth under control and stress condition. MG levels were estimated in all of them and MG levels of mutants were compared with their complemented counterpart.

**Figure 3:**
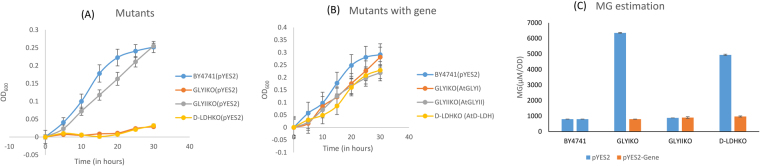
Stress tolerance assay in yeast mutants of MG detoxification enzymes in salinity stress: BY4741 wild type yeast cells, GLYIKO, GLYIIKO and D-LDHKO mutant yeast cells containing empty vector pYES2 were grown in presence of salinity stress (**A**). These mutant cells were transformed with their respective mutant genes cloned in pYES2 vector and the complemented yeast cells were grown in presence of salinity stress and their growth was monitored along with wild type BY4741 cells (**B**). MG levels were estimated in all of these samples after 30 hours and contribution of these genes in detoxification of MG was compared (**C**).

In salinity stress, BY4741 and GLYIIKO cells grew but the GLYIKO and D-LDHKO cells did not grow (Fig. 3A). Absence of GLYI and D-LDH genes from the cells ceased cell growth in saline conditions. When these cells were complemented, GLYI and D-LDH complemented mutants grew similar to the wild type cells (Fig. 3B). On comparing the MG levels of the mutant cells with the complemented cells, it has been observed that GLYIKO and D-LDHKO cells have extremely high MG whereas GLYIIKO has MG levels similar to wild type. After complementation with respective genes in the mutants, MG level in the GLYIKO and D-LDHKO cells were restored to lower level and that too almost equal to the wild type cells (Fig. 3C).

### D-LDH and GLYI are majorly responsible for conferring oxidative stress tolerance in yeast cells

**Figure 4:**
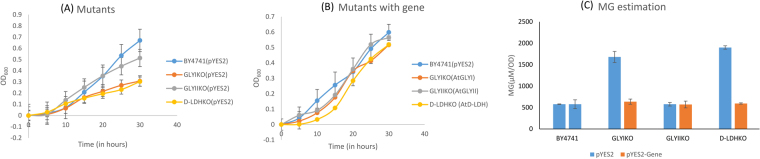
Heterologous expression of MG detoxifying enzymes in yeast mutants confers oxidative stress tolerance: BY4741 wild type yeast cells, GLYIKO, GLYIIKO and D-LDHKO mutant yeast cells containing empty vector pYES2 were grown in presence of oxidative stress (**A**). These mutant cells were transformed with their respective mutant genes cloned in pYES2 vector and the complemented yeast cells were grown in presence of oxidative stress and their growth was monitored along with wild type BY4741 cells (**B**). MG levels were estimated in all of these samples after 30 hours and contribution of these genes in detoxification of MG was compared (**C**).

In oxidative stress, D-LDH was found to be the most important MG detoxifying gene candidate. The presence of H_2_O_2_ in the media severely affected the growth of D-LDHKO cells (Fig. 4A). GLYIKO cells also showed a slow growth pattern however GLYIIKO cells maintained a steady growth comparable to the wild type yeast cells. The MG level of D-LDHKO cells was recorded to be the highest followed by GLYIKO cells which accumulated a little less MG than D-LDHKO cells (Fig. 4C). However, the MG level of GLYIIKO cells was similar to the BY4741 cells. The complemented D-LDHKO and GLYIKO cells were comparable to wild type cells in terms of their growth (Fig. 4B). The growth of complemented GLYIIKO cells was also similar to the wild type cells. The complementation of mutants with GLYI and D-LDH genes reduced the MG to much lower level. The MG level of GLYIIKO and wild type cells was higher than the complemented GLYIKO and D-LDHKO cells (Fig. 4C).

### GLYI provides more tolerance in exogenous MG stress in yeast cells

**Figure 5:**
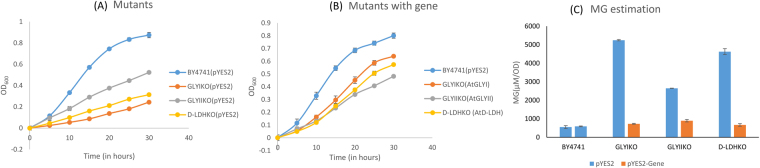
Stress tolerance assay in yeast mutants of MG detoxification enzymes in MG detoxification: BY4741 wild type yeast cells, GLYIKO, GLYIIKO and D-LDHKO mutant yeast cells containing empty vector pYES2 were grown in presence of exogenous MG (**A**). These mutant cells were transformed with their respective mutant genes cloned in pYES2 vector and the complemented yeast cells were grown in presence of exogenous MG stress and their growth was monitored along with wild type BY4741 cells (**B**). MG levels were estimated in all of these samples after 30 hours and contribution of these genes in detoxification of MG was compared.

In response to exogenous MG stress, wild type cells and GLYIIKO cells grew better than the D-LDHKO cells. GLYIKO cells showed negligible growth (Fig. 5A). The level of MG in GLYIKO cells was highest followed by D-LDHKO cells, GLYIIKO cells and BY4741 cells. After complementation, the growth scenario just reversed with GLYI cells growing the best followed by D-LDH, GLYII and BY4741 cells (Fig. 5B). GLYI complemented mutants accumulated lowest MG level followed by D-LDH and GLYII complemented cells. Wild type cells had the highest MG than any of the complemented mutant cells (Fig. 5C). GLYI and D-LDH thus are key players in MG detoxification.

### GLYI is the most important enzyme for MG detoxification

**Figure 6:**
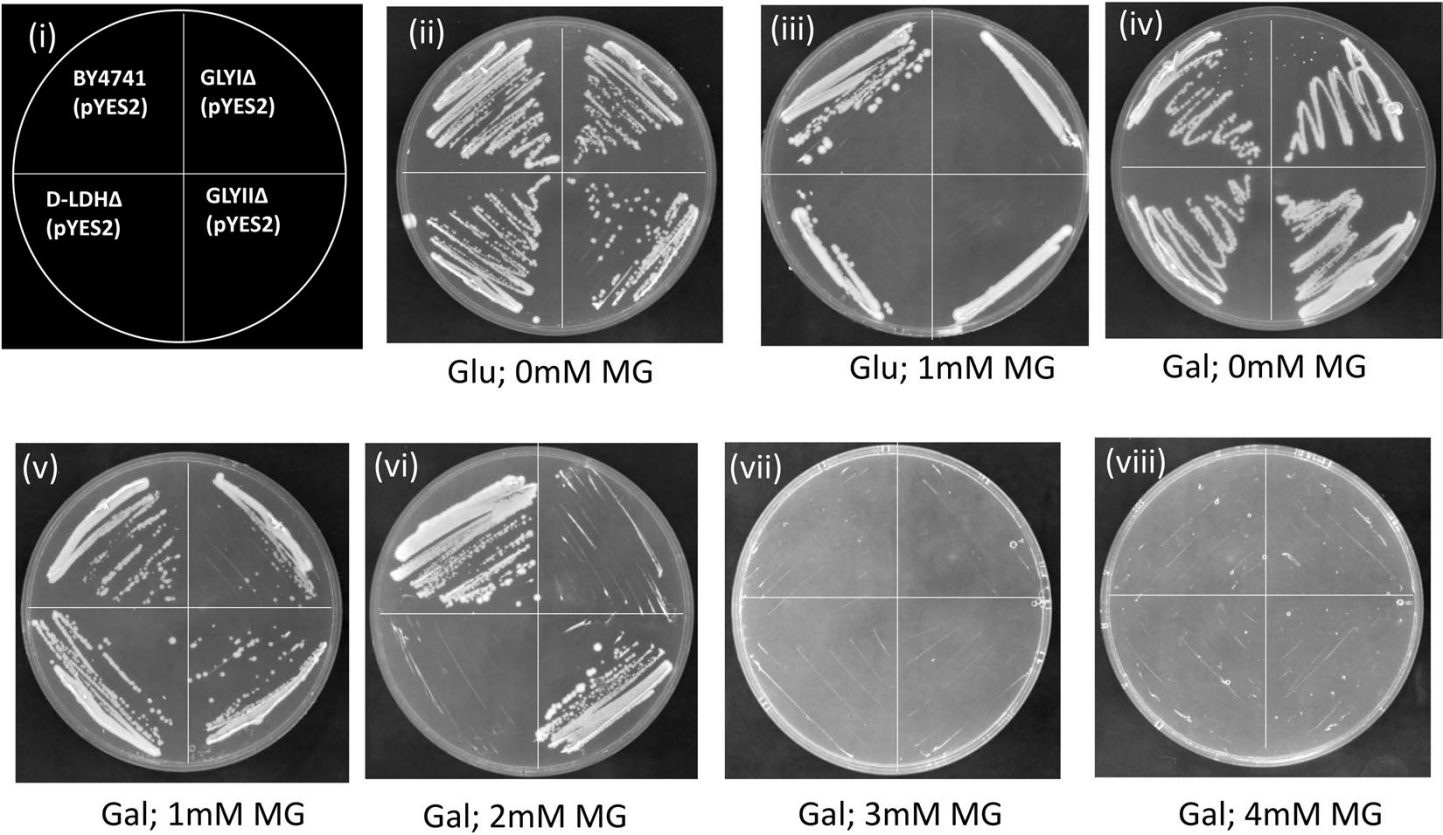
Loss of function study reveals role of different genes in MG detoxification: BY4741 wild type yeast cells, GLYIKO, GLYIIKO and D-LDHKO mutant yeast cells containing empty vector pYES2 were grown on Ura^−^ SD agar plates with different supplements to check for their contribution to MG detoxification. (**i**) Pictorial representation of various strains used. (**ii**) Medium supplemented with 20% glucose. (**iii**) medium supplemented with 20% glucose and 1 mM MG. **(iv**) Medium supplemented with 20% galactose. (**v**) Medium supplemented with 20% galactose and 1 mM MG. (**vi**) Medium supplemented with 20% galactose and 2 mM MG. (**vii**) Medium supplemented with 20% galactose and 3 mM MG. (**viii**) Medium supplemented with 20% galactose and 4 mM MG.

**Figure 7:**
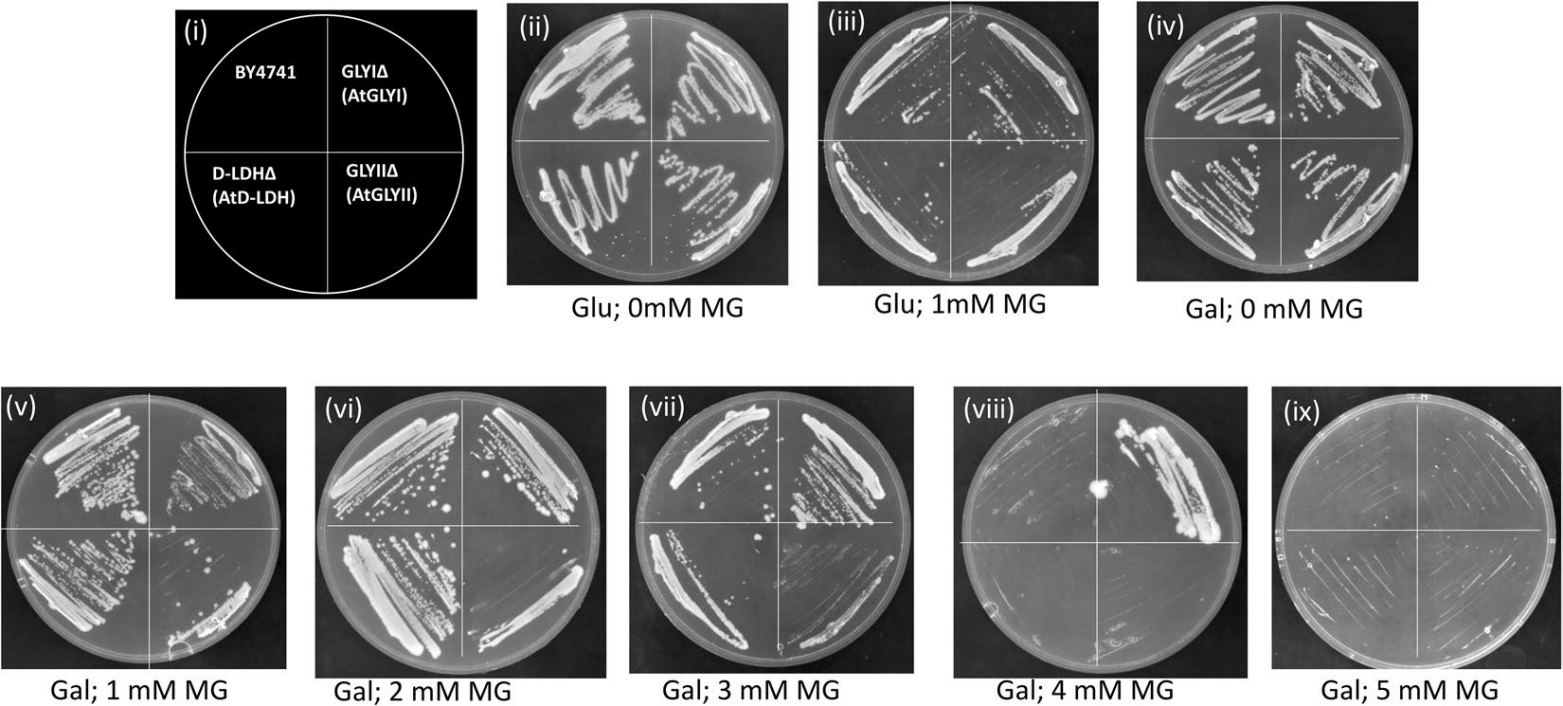
Functional complementation of MG detoxification genes in respective yeast mutant confirms the contribution of different genes in MG detoxification: Yeast cells mutant of GLYI, GLYII and D-DLH were transformed with pYES2-AtGLYI, pYES2-AtGLYII and pYES2-AtD-LDH respectively and were grown in Ura^−^ SD solid media with different supplements to check for their contribution to MG detoxification. Wild type BY4741 cells containing empty vector pYES2 were used for comparison. (**i**) Pictorial representation of various strains used. (**ii**) Medium supplemented with 20% glucose. (**iii**) medium supplemented with 20% glucose and 1 mM MG. (**iv**) Medium supplemented with 20% galactose; (**v**) Medium supplemented with 20% galactose and 1 mM MG. (**vi**) Medium supplemented with 20% galactose and 2 mM MG. (**vii**) Medium supplemented with 20% galactose and 3 mM MG. (**viii**) Medium supplemented with 20% galactose and 4 mM MG. (**ix**) Medium supplemented with 20% galactose and 5 mM MG.

BY4741, GLYIKO, GLYIIKO and D-LDHKO cells containing empty vector were grown on Ura^−^ SD agar plates with either glucose or galactose and different concentrations of MG. In the absence of MG, growth was seen in all the cells with both glucose as well as galactose in the media. GLYIKO and D-LDHKO cells could not grow at more than 1 mM exogenous MG concentration. GLYIIKO and wild type cells grew till 2 mM MG but did not grow on plates with 3 mM MG (Fig. 6). All the complemented cells were able to grow in media supplemented with both glucose as well as galactose in absence of MG. The complemented GLYIKO cells could grow upto 4 mM MG, AtD-LDH complemented D-LDHKO cells grew upto 3 mM MG; AtGLYII complemented GLYIIKO cells grew upto 2 mM MG and wild type cells showed growth till 3 mM MG concentration (Fig. 7).

## Discussion

Methylglyoxal is a cytotoxic by product of various metabolic reactions^24^. It reacts with the major macromolecules of the cell and forms adducts and is detrimental to the system. In abiotic stress conditions, MG level increases by 4–5 folds and its toxic effects are also accentuated. Therefore, it is important to detoxify MG from the cell. Many enzymes and pathways are known to participate in detoxification of MG^33–39^. Glyoxalase system constitutes the major MG detoxifying pathway and it is composed of two enzymes; GLYI and GLYII. Recently, another enzyme D-LDH has been linked with MG detoxification^31,32^. These three enzymes work together and convert MG into D-pyruvate which enters into TCA cycle and thus the toxic metabolite MG is used for energy production. There have been many studies involving characterization of these enzymes as well as overexpressing these enzymes for engineering stress tolerance though much is not known about the comparative role of each of these genes in MG detoxification.

This study was aimed at identifying the key MG detoxifying enzyme(s) amongst GLYI, GLYII and D-LDH. Loss of function and overexpression was attempted in *E. coli* and yeast to comment on contribution of each of these genes in providing tolerance towards different abiotic stresses. Out of the multimember gene families of GLYI, GLYII and D-LDH from Arabidopsis, the most efficient member of each was chosen. The chosen AtGLYI, AtGLYII and AtD-LDH genes were cloned in pJET, subcloned in pET28a bacterial expression vector as well as in pYES2 yeast expression vector. All the three genes were overexpressed in BL21 *E. coli* cells in presence of different abiotic stresses and their growth pattern was compared with BL21 cells containing empty vector pET28a. Their MG levels were also estimated and compared. The cells overexpressing the MG detoxification enzymes were able to detoxify the increased MG and thus, helped the cells to grow in stress conditions. The cells that exhibit better growth detoxified more MG consequently showing lower MG level. The MG levels indirectly indicate the importance of each of these enzymes in MG detoxification under stress conditions. In all the stresses, *E. coli* cells overexpressing MG detoxification enzymes showed better growth than cells with empty vector. In saline stress, GLYI overexpressing cells grew the best followed by cells overexpressing GLYII, D-LDH and cells with empty vector (Fig. 2A). The MG level was least in GLYI overexpressing cells, followed by GLYII and D-LDH expressing cells (Fig. 2D). This suggested that GLYI is more efficient than GLYII and D-LDH in salinity stress conditions. In oxidative stress, D-LDH followed by GLYI emerged as the most important enzymes as the cells overexpressing them showed better growth among the three genes and their MG level was also considerably less (Fig. 2B and E). However, in conditions of exogenous MG, GLYI overexpressing cells were most efficient in detoxifying the MG and they showed better growth than all other cells (Fig. 2C). The MG level of GLYI expressing cells was lowest followed by GLYII and D-LDH expressing cells (Fig. 2F). The MG detoxification by these enzymes in case of exogenous MG stress is related directly to their enzymatic properties such as their catalytic efficiency (Kcat/Km ratio). The Kcat/Km ratio of AtGLYI, AtGLYII and AtD-LDH is 174.9 × 10^6^ M^−1^ s^−1^, 4.7 × 10^6^ M^−1^ s^−1^, 13.81 × 10^6^ M^−1^ s^−1^, respectively^31,41,42^. The catalytic efficiency of these enzymes follows the same order as their MG detoxification capability; GLYI being the most efficient followed by D-LDH and then, GLYII.

The GLYIKO, GLYIIKO and D-LDHKO cells complemented with AtGLYI, AtGLYII and AtD-LDH, respectively along with their complemented counterparts were grown in presence of different abiotic stresses and their growth was observed. Wild type BY4741 cells were used as a positive control as they possess all the three genes. All these cells were monitored for their growth over the span of 30 hours. Absence of GLYI and D-LDH showed severe effects in response to salinity stress as the cells mutant for these genes did not show any growth but the growth of GLYIIKO yeast cells was similar to wild type cells (Fig. 3A). GLYIKO and D-LDHKO cells accumulated quite higher level of MG as compared to GLYIIKO and the wild type cells. After complementation with the genes, MG levels were restored to level comparable to wild type cells. Complementation of GLYIKO cells with AtGLYI reduced the MG levels from ~6 to ~1 mM/OD and presence of AtD-LDH in D-LDHKO cells reduced MG from ~5 to ~1 mM/OD (Fig. 3C). Therefore, GLYI is the most important gene for providing tolerance in salinity stress. D-LDH also has a role in providing salinity tolerance but lesser than GLYI.

In oxidative stress, D-LDH seems the most promising candidate gene for providing tolerance. D-LDHKO and GLYIKO cells showed very less growth in comparison to GLYIIKO and the wild type cells when grown in presence of H_2_O_2_ in the media (Fig. 4A). After complementing the mutants with the respective genes, the growth pattern just reversed with the AtD-LDH and AtGLYI complemented mutant cells growing much better than the complemented GLYIIKO cells and similar to the wild type cells (Fig. 4B). The MG levels of D-LDHKO cells were highest followed by GLYIKO. GLYIIKO and wild type cells again showed similar MG levels. Even after complementing the GLYIIKO cells with AtGLYII, MG levels were as before complementation and equal to the wild type cells. However, functional complementation of D-LDHKO with AtD-LDH gene led to ~3.2 fold decrease in MG levels and AtGLYI complementing the GLYIKO reduced MG level by ~2.5 folds (Fig. 4C). This experiment shows D-LDH to be the most important candidate in providing oxidative stress tolerance followed by GLYI.

The emergence of D-LDH as the key enzyme in providing oxidative stress tolerance in both *E. coli* and yeast was quite surprising. Probably by some other mechanism, D-LDH works to mitigate oxidative insult inflicted on the cells. The major effect of oxidative stress is overproduction of ROS and the major intracellular target of oxidative stress is mitochondria. The localization of D-LDH in mitochondria possibly favors the cell in protection against oxidative damage. D-LDH converts D-lactate into pyruvate which has multiple antioxidative roles^44^. Pyruvate embodies antioxidant properties due to its α-keto carboxylate structure which enables it to directly and non-enzymatically neutralize peroxides and peroxynitrites^45^. H_2_O_2_ is the major source of oxidative stress. Pyruvate forms acetate, carbon-dioxide and water by reacting with H_2_O_2_. Also, pyruvate increases the NADPH/NADP+, the source of reducing power which enables Glutathione reductase to maintain GSH/GSSG ratio. Thus, pyruvate restores the redox potential of GSH, the major antioxidant by providing NADPH reducing equivalents. This occurs by two different mechanisms^45^. Pyruvate carboxylation by certain enzymes such as pyruvate carboxylase and malic enzyme leads to formation of citrate which moves to cytosol where citrate suppresses the activity of enzyme phosphofructokinase and diverts Glucose-6-phosphate to HMP pathway; thereby increasing NADPH formation. Also, citrate acts as the substrate for the NADP+ dependent enzyme isocitrate dehydrogenase. This increased NADPH is the prime requirement of enzymatic antioxidant defense system to function properly. Pyruvate is capable of doing this both intracellularly as well as extracellularly as it is the sole ketoacid that is secreted. These antioxidative protective effects of pyruvate have been investigated both *in vitro* and *in vivo* in different cell types (cardiomyocytes, neural cells) and whole organs (liver, Kidney) but not so well characterized in plants. If D-LDH is not present, D-lactate will accumulate in the cells which is toxic for the system. Firstly, lactate has been found ineffective in reacting with H_2_O_2_ and protecting against H_2_O_2_ induced toxicity. Secondly, presence of lactate in the media affects seedling development in *Arabidopsis thaliana* negatively whereas plants overexpressing D-LDH were found to detoxify D-lactate to pyruvate and survive^46^. Thus, undeniably by converting toxic lactate into pyruvate, D-LDH confers oxidative stress tolerance.

When these mutant cells were grown in media containing exogenous MG, wild type cells showed maximal growth followed by GLYIIKO cells, D-LDHKO cells and GLYIKO cells. In presence of MG, the growth of GLYIIKO cells was lesser than the wild type cells; so GLYII has a definite role in detoxification of MG (Fig. 5A). Absence of GLYI has the major effects on cell growth and so it is the most important gene for MG detoxification. The MG levels of GLYIKO cells were the highest followed by D-LDHKO and the GLYIIKO cells (Fig. 5C). Wild type cells had all the genes for MG detoxification, so they had the lowest MG levels in comparison to the mutants. When the complemented mutant cells were grown in different stress conditions, GLYI complemented cells showed more tolerance followed by D-LDH complemented cells and the GLYII complemented cells (Fig. 5B). Wild type cells grew the least in this case. But the presence of AtGLYI and AtD-LDH both reduced the MG levels by 5 folds. On complementation of GLYIIKO cells with AtGLYII gene, MG levels decreased by ~3 folds (Fig. 5C). So, GLYII gene plays an important role in MG detoxification but GLYI and D-LDH are more important than GLYII. Surprisingly, the MG levels of D-LDHKO were high and almost equal to the GLYIKO cells. Earlier, similar results have been found where silencing of D-LDH caused changes in GLYI and aldo-keto reductase behavior^47^. As D-lactate knockdown affects the activity of both glyoxalase system and aldo-keto reductases, it causes increment in the MG level of D-LDHKO lines.

Further, to validate the effect of each gene in MG detoxification, mutant as well as complemented cells were streaked on solid media containing either glucose or galactose with different concentration of MG (0 mM- 5 mM). On glucose as well as galactose plates having no MG, all the cells grew well. In presence of 1 mM MG and glucose, only the wild type cells grew and the mutants did not grow well. In presence of galactose in the plates, GLYIKO cells could not grow well even in 1 mM MG concentration whereas all other cells showed a steady growth (Fig. 6). In 2 mM MG, D-LDHKO cells ceased to grow but GLYIIKO and BY4741 cells were able to grow well. Therefore, D-LDH is the second important gene for MG detoxification. In presence of 3 or 4 mM MG, none of the cells were able to grow.

In other case, the mutant cells were complemented with respective gene from *Arabidopsis thaliana*. All the complemented cells along with wild type cells were grown on plates with different MG concentrations. In plates having no MG, all the cells grew well in presence of both glucose and galactose. None of the cells grew well in plates with 1 mM MG and glucose. In presence of galactose and 1 mM and 2 mM MG, all the cells showed normal growth. AtGLYII complemented GLYIIKO cells did not grow on plate with 3 mM MG. The GLYIKO cells which did not grow on more than 1 mM MG, after complementation with AtGLYI grew till 4 mM MG. Similarly after complementation, the D-LDHKO cells showed growth upto 3 mM MG (Fig. 7). This implies AtGLYI to be the most important gene for MG detoxification. AtD-LDH is less efficient for MG detoxification as compared to AtGLYI but much better than AtGLYII. This also correlates with the catalytic efficiency of these enzymes^31,41,42^.

This is the first study that compares the efficiency of different enzymes of MG detoxification. Based on growth curve analysis as well as streaking using wild type, mutant as well as complemented strains of both *E. coli* and yeast, it was found that GLYI and D-LDH are the key players in MG detoxification and abiotic stress tolerance. SLG, an end-product of MG detoxification through GLYI, would be accumulated in higher concentration in cells if GLYII is mutated. While mutation in GLYI and D-LDH accumulation would lead to higher accumulation of MG and D-lactate, respectively. Based on the results obtained, we hypothesize MG and D-Lactate are more cytotoxic intermediates compared to SLG as reflected by negligible effect of GLYII mutation in stress response of both *E. coli* and yeast. Here, we have shown D-LDH to be an integral part of MG detoxification and a critical enzyme in abiotic stress tolerance. D-LDH converts the toxic metabolite D-lactate to pyruvate which has multiple protective functions and thereby completes the cycle of MG detoxification. Earlier studies have shown that overexpressing both GLYI and GLYII could provide better abiotic stress tolerance compared to GLYI/GLYII alone^48,49^. This study paves a new direction for crop engineering, indicating engineering D-LDH along with GLYI and GLYII might be able to surpass the stress mitigating effect of GLYI/GLYII double transgenics. We are currently working on GLYI, GLYII and D-LDH mutants of Arabidopsis which would provide more insights about the importance of these enzymes in plant system.

## Electronic supplementary material

Supplementary Information
