# Supplementary material for: GLYI and D-LDH play key role in methylglyoxal detoxification and abiotic stress tolerance
Source: Sci Rep. 2018 Apr 3;8:5451. doi: 10.1038/s41598-018-23806-4 (PMC5883029; doi:10.1038/s41598-018-23806-4)
Supplement: Supplementary file 1 — Supplementary Information [file 41598_2018_23806_MOESM1_ESM.pdf]

GLYI and D-LDH play key role in methylglyoxal detoxification and abiotic stress tolerance

Muskan Jain[1], Preeti Nagar[1], Ayush Sharma[1], Rituraj Batth[1], Sakshi Aggarwal[1], Sumita Kumari[2], Ananda Mustafiz[1*]

[1] Faculty of Life Sciences and Biotechnology, Laboratory of Plant Molecular Biology, South Asian University, Akbar Bhawan, Chanakyapuri, New Delhi 110021, India

[2]School of Biotechnology, Sher-e-Kashmir University of Agricultural Sciences and Technology, Jammu 180009, India

[*] Corresponding author

Email: amustafiz@sau.ac.in

Supplementary Table S1: A list of primers used in the study

S. No.	Primer Name	Primer Sequence
1.	AtGLYI EcoRI-F	GGAATTCATGGCGTCGGAAGCGAGGGAATC
2.	AtGLYI XhoI-R	CCGCTCGAGTCAAGCTGCGTTTACGGTAGTAGTT
3,	AtGLYII EcoRI-F	GGAATTCATGAAGATCTTCCACGTTCCTTGTCTG
4.	AtGLYII XhoI-R	CCGCTCGAGTCATCCCCTCCACTGATCCTTCTT
5.	AtD-LDH EcoRI-F	GGAATTCATGGCTTTCGCTTCAAAATTCGCT
6.	AtD-LDH NotI-R	TTGCGGCCGCTTAGAAACATACATGAGGAGGAATTAAC
